# Spectral Characterization and Molecular Dynamics Simulation of Pesticides Based on Terahertz Time-Domain Spectra Analyses and Density Functional Theory (DFT) Calculations

**DOI:** 10.3390/molecules23071607

**Published:** 2018-07-02

**Authors:** Fangfang Qu, Lei Lin, Yong He, Pengcheng Nie, Chengyong Cai, Tao Dong, Yi Pan, Yu Tang, Shaoming Luo

**Affiliations:** 1 College of Biosystems Engineering and Food Science, Zhejiang University, Hangzhou 310058, China; ffqu@zju.edu.cn (F.Q.); linlei2016@zju.edu.cn (L.L.); yhe@zju.edu.cn (Y.H.); alayy@zju.edu.cn (C.C.); dt2016@zju.edu.cn (T.D.); 2 Key Laboratory of Spectroscopy Sensing, Ministry of Agriculture, Hangzhou 310058, China; 3 State Key Laboratory of Modern Optical Instrumentation, Zhejiang University, Hangzhou 310027, China; 4 Laser Information Technology Research Center, Harbin Institute of Technology Shenzhen Graduate School, Guangdong 518055, China; panyi@huaxunchina.cn; 5 College of Automation, Zhongkai University of Agriculture and Engineering, Guangzhou 510225, China; tangyu_mycauc@163.com (Y.T.); smluo@gdut.edu.cn (S.L.)

**Keywords:** terahertz time-domain spectroscopy, wavelet threshold de-noising, spectral baseline correction, density functional theory, pesticides, molecular dynamics simulations

## Abstract

This work provides the experimental and theoretical fundamentals for detecting the molecular fingerprints of six kinds of pesticides by using terahertz (THz) time-domain spectroscopy (THz-TDS). The spectra of absorption coefficient and refractive index of the pesticides, chlorpyrifos, fipronil, carbofuran, dimethoate, methomyl, and thidiazuron are obtained in frequencies of 0.1–3.5 THz. To accurately describe the THz spectral characteristics of pesticides, the wavelet threshold de-noising (WTD) method with db 5 wavelet fucntion, 5-layer decomposition, and soft-threshold de-noising was used to eliminate the spectral noise. The spectral baseline correction (SBC) method based on asymmetric least squares smoothing was used to remove the baseline drift. Spectral results show that chlorpyrifo had three characteristic absorption peaks at 1.47, 1.93, and 2.73 THz. Fipronil showed three peaks at 0.76, 1.23, and 2.31 THz. Carbofuran showed two peaks at 2.72 and 3.06 THz. Dimethoate showed three peaks at 1.05, 1.89, and 2.92 THz. Methomyl showed five peaks at 1.01, 1.65, 1.91, 2.72, and 3.20 THz. Thidiazuron showed four peaks at 0.99, 1.57, 2.17, and 2.66 THz. The density functional theory (DFT) of B3LYP/6-31G+(d,p) was applied to simulate the molecular dynamics for peak analyzing of the pesticides based on isolated molecules. The theoretical spectra are in good agreement with the experimental spectra processed by WTD + SBC, which implies the validity of WTD + SBC spectral processing methods and the accuracy of DFT spectral peak analysis. These results support that the combination of THz-TDS and DFT is an effective tool for pesticide fingerprint analysis and the molecular dynamics simulations.

## 1. Introduction

As a novel analytical technique, terahertz (THz) spectroscopy has multivariate properties such as fingerprint absorption, penetration, coherence, transient and low ionization damage [1,2]. It has been demonstrated many valuable and versatile applications in the detection of optical properties of many dielectrics and biochemical materials [3,4]. Intermolecular interactions, skeleton vibrations, oscillations, and the rotational transitions of different vibrational frequencies result in the distinct fingerprints in the THz region (0.1–10 THz) [5,6]. THz spectroscopy is sensitive to materials with different chemical composition or crystal structure. Therefore, it can be used to characterize the specific spectral information of the materials [7,8]. It provides useful information for the analysis of materials requiring accurate identification and detection [9]. However, the interpretation and understanding of THz spectra is still a challenge at present [10]. The inevitable systematic and random errors of THz spectrometer have a negative influence on the optical properties characterization of materials [11]. The spectral noise and baseline drifts will deteriorate the qualitative or quantitative analytical results in multivariate analysis [12,13]. Therefore, it is necessary to eliminate the influencing factors and improve spectra signal-to-noise ratio (SNR) before characterizing the optical properties of the measured materials [14]. Based on the processed high quality spectra, the assignments of THz spectra are typically required to study the formation mechanism of the experimental absorption peaks to specific modes. The density functional theory (DFT) is a powerful tool to investigate the molecular structures and harmonic vibrational frequencies of molecules [15,16]. It has been proven to be capable of predicting the assignment of THz spectra with specific absorption peaks [17].

Pesticides are chemically synthesized compounds of several biological macromolecules, which can be used to control grasses, diseases, harmful insects, and pests that endanger agriculture and forestry [18,19]. In addition, they can also be used to purposefully regulate, control, and affect the metabolism, growth, development, and reproduction of plants [20,21]. However, serious issues of food safety and environmental pollution have attracted more and more attention due to the misuse of these toxic residue pesticides [22,23]. Hence, it is of great significance for the detection of pesticides. In the previous work, the pesticides were detected by enzyme-linked immunosorbent assay (ELISA), high performance liquid chromatography (HPLC), gas chromatography (GC), and ultra performance liquid chromatography-mass spectrometry (UPLC-MS) [24,25]. These methods are usually time-consuming and complicated for sample preparation [26,27]. Hua et al. studied the methods of qualitative and quantitative detection of pesticides (0.5–1.6 THz) using THz time-domain spectroscopy (THz-TDS) [28]. Lee et al. detected pesticide residues (0.5–2.0 THz) using THz near field enhancement [29]. These researches were remarkable in pesticides detection based on THz technology. However, the spectra were obtained only in a narrow frequency range, resulting in some absorption features beyond this range could not be detected.

In this work, the spectral characteristics of six kinds of pesticides, including chlorpyrifos, fipronil, carbofuran, dimethoate, methomyl, and thidiazuron, were investigated by using THz time-domain spectroscopy (THz-TDS). First, to improve the spectral SNR, the wavelet threshold de-noising (WTD) method was used to remove the THz spectral noises. Then, the spectral baseline correction (SBC) method was used to remove the baseline drift caused by the high frequency absorption. Finally, the DFT method was used to investigate the molecular geometric configuration and vibration modes assignment of pesticide samples. The absorption peaks of the experimental spectra were analyzed based on the theoretical calculation results of DFT molecular dynamics simulations. This work was presented to study the THz spectral processing methods and to analyze the characteristic absorption peaks of pesticides. It was aimed to provide the theoretical and experimental basis for the detection of pesticides by using THz-TDS.

## 2. Results and Discussion

### 2.1. THz Time-Domain Waveforms and Frequency-Domain Spectra

**Figure 1:**
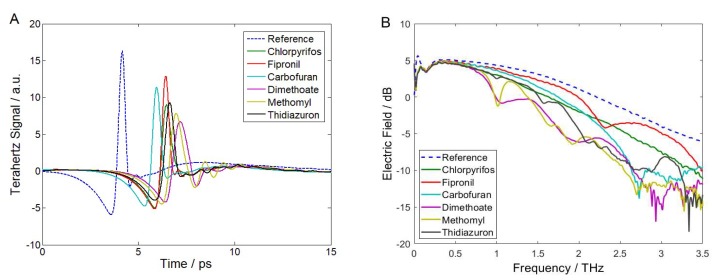
Terahertz (THz) spectra of the references and pesticide samples. (**A**) Time-domain waveforms and (**B**) Frequency-domain spectra.

The THz spectra of polyethylene (PE) and pesticide samples are measured by THz-TDS as the reference and signal spectra, respectively. Figure 1 shows the time-domain waveforms and frequency-domain spectra of the references and the six kinds of pesticide samples. The fully recorded THz pulse time trace is 0–33.5 ps. To present a better visibility of the time-domain waveforms, the THz pulse time trace in the range of 0–15 ps is showed in Figure 1A. It is seen in that the amplitude of the six pesticides is attenuated and the time is delayed compared with the reference. The amplitude of the reference is 16.325 a.u. The amplitude of chlorpyrifos, fipronil, carbofuran, dimethoate, methomyl, and thidiazuron dropped to 9.016, 12.924, 11.417, 6.737, 7.884, and 9.296 a.u, respectively. This is due to the absorption, reflection, and scattering of THz waves by the pesticide samples. The time delay of the reference is 4.169 ps. Compared with the reference, the time delay of the six pesticide samples are 6.445, 6.413, 5.962, 7.166, 6.961 and 6.650 ps, respectively. This is related to the differences of THz wave propagation velocity in PE and pesticide samples. The frequency-domain spectra in logarithmic (dB) scale obtained by Fourier transform of the corresponding time-domain spectra (0–33.5 ps) are shown in Figure 1B. The electric field of the pesticide samples is weakened compared with the reference. This is caused by the sample absorption of THz wave. The results show that there are anomalous spectral pits in different frequency regions of the pesticide samples, but there is no such phenomenon shown in the reference spectrum. It implies that PE is insensitive to THz wave and has no interference to the samples. Therefore, these anomalous spectral pits can be assigned as the specific THz optical characteristics of the pesticides.

### 2.2. Analysis of Absorption and Refraction Characteristics

**Figure 2:**
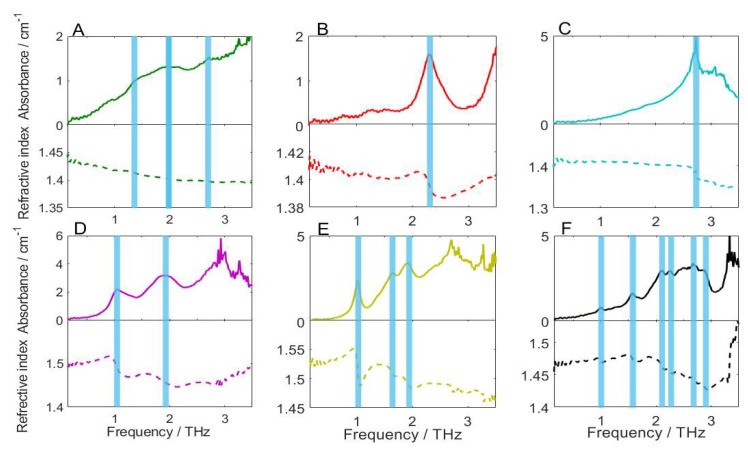
The absorption and refraction spectra of six pesticide samples. (**A**) chlorpyrifos, (**B**) fipronil, (**C**) carbofuran, (**D**) dimethoate, (**E**) methomyl, and (**F**) thidiazuron.

In order to analyze the absorption and refraction characteristics of the pesticides, the THz absorption and refraction spectra were calculated based on the frequency dependent Fresnel formulas. Figure 2 depicts the absorption coefficients and refractive indices of the six pesticide samples from 0.1 to 3.5 THz. It can be observed that the baselines of the absorption spectra raise with the increase of frequency. This may be due to the higher frequency enables the stronger absorption and scattering of THz wave by the sample, leading to the gradual upward drift of the baseline. Furthermore, the spectral SNR is low, which is probably interfered by high frequency oscillation noise, low frequency instrument error noise, and sampling multiple reflection noise. Results show that chlorpyrifos had three weak absorption peaks at 1.47, 1.93, and 2.73 THz, respectively. Fipronil and carbofuran showed sharp absorption peaks at 2.31 and 2.72 THz, respectively. Dimethoate showed two broad absorption peaks near 1.05 and 1.89 THz. Methomyl had a sharp absorption peak at 1.01 THz, and two weak absorption peaks at 1.65 and 1.91 THz. Thidiazuron showed six absorption peaks at 0.99, 1.57, 2.10, 2.25, 2.51, and 2.66 THz. However, some fake peaks may appear and some true peaks may be submerged under the influence of noise. Besides this, some spectral disturbances can be observed in these spectra, but further analysis is needed to determine whether there are other absorption peaks. These factors will affect the analytical accuracy of the absorption peaks. Therefore, it is necessary to preprocess the THz spectra to improve the spectral quality.

The average refractive index of chlorpyrifos, fipronil, carbofuran, dimethoate, methomyl, and thidiazuron in frequencies of 0.1–3.5 THz is 1.409, 1.400, 1.394, 1.475, 1.503, and 1.464, respectively. It reflects the dielectric constant, absorption, and dispersion characteristics of the pesticide. As can be seen in Figure 2, there is a change in refractive index at the location of each characteristic absorption peak. This phenomenon indicates that there is an abnormal dispersion near the absorption peak. When the judgement of the absorption peaks is ambiguous due to noise or weak spectral intensity, the change properties of refractive index can be used to assist with the determination of the true absorption peaks.

### 2.3. Spectral De-noising and Baseline Correction

To improve the analysis precision of the THz spectra, WTD method was applied for THz spectral de-noising, which is conducted in the wavelet toolbox in Matlab 2014 software (MathWorks, Natick, MA, USA). The three parameters of WTD were set as: wavelet function is db5, decomposition lever is 5, threshold function is soft-threshold. Table 1 lists the determined thresholds of each decomposition level and the evaluations of WTD de-noising effectiveness. These thresholds are used to filter the wavelet coefficients of the noise. Furthermore, they are adjustable in the toolbox to control the de-noising effect of the final processed spectrum. The main peak of chlorpyrifos, fipronil, carbofuran, dimethoate, methomyl, and thidiazuron was located at 1.99, 2.31, 2.72, 2.92, 2.72, and 2.66 THz, respectively. The calculated peak SNR (PSNR) of the pesticide main peak is relatively high. In addition, the root mean squares error (RMSE) between the original and the de-noised spectrum is relatively low. These results indicate that WTD is effective for removing spectral noise.

**Figure 3:**
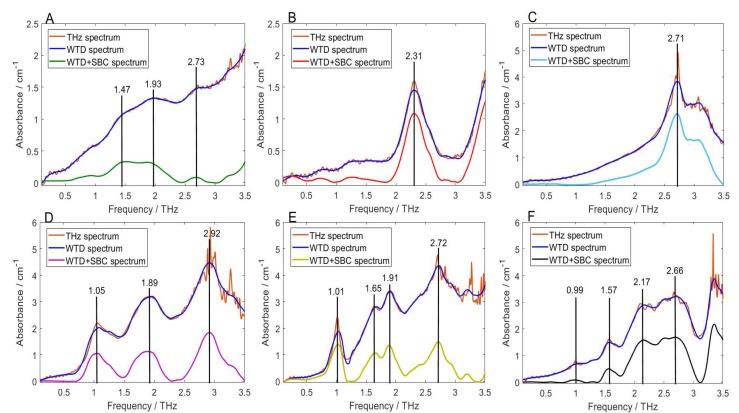
The original, de-noised, and baseline corrected spectra of the six pesticide samples. (**A**) chlorpyrifos; (**B**) fipronil; (**C**) carbofuran; (**D**) dimethoate; (**E**) methomyl, and (**F**) thidiazuron.

Figure 3 plots the original, de-noised, and baseline corrected spectra of the six pesticide samples. For the low frequency (0.1–0.7 THz) instrument errors and the high frequency (3–3.5 THz) oscillation noises, the spectral de-noising effect is particularly obvious. For the absorption peaks of the six pesticides in the original THz spectra, WTD can effectively remove the noise while maintaining the original peak shape and peak position. In addition, some new absorption peaks were found at high frequency regions (3–3.5 THz) after being processed by WTD. However, their formation mechanisms need to be further analyzed to determine the authenticity of the peaks. SBC is then applied to the spectra processed by WTD. The parameters were set as: regularization parameter *μ* = 100, weight parameter *p* = 0, and spectral length *n* = 463. The results show that the peak location remains unchanged and the peak shape is more prominent, which indicates that SBC is effective in correcting the baseline drifts of THz spectra.

### 2.4. Molecular Dynamics Simulations by DFT

#### 2.4.1. Molecular Geometric Configuration

**Figure 4:**
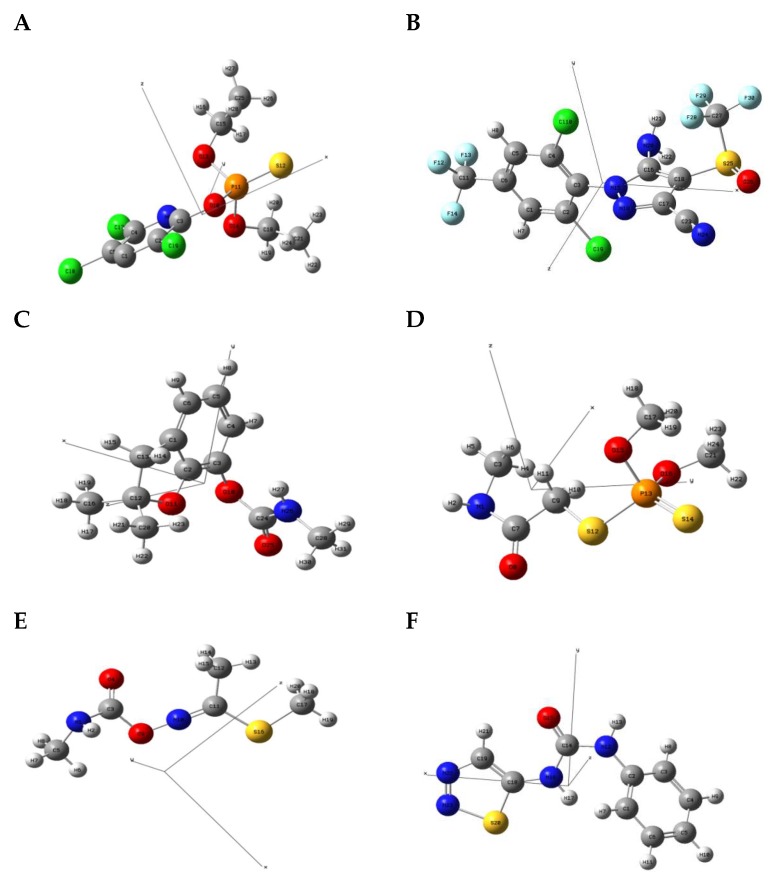
The molecular geometric configuration of the pesticide isolated molecule. (**A**) chlorpyrifos, (**B**) fipronil; (**C**) carbofuran; (**D**) dimethoate; (**E**) methomyl, and (**F**) thidiazuron.

The geometry structure of the isolated molecule of the six pesticide compounds, including chlorpyrifos (C_9_H_11_Cl_3_NO_3_PS), fipronil (C_12_H_4_Cl_2_F_6_N_4_OS), carbofuran (C_12_H_15_NO_3_), dimethoate (C_5_H_12_NO_3_PS_2_), methomyl (C_5_H_10_N_2_O_2_S), and thidiazuron (C_9_H_8_N_4_OS), were calculated and tightly optimized using the hybrid functional model of B3LYP Becke with 6-31G+(d,p) basis set (Lee-Yang-Parr functional) in Gaussian 2016 software (Gaussian Inc., Wallingford, CT, USA). These optimized molecule structures were drawn in Gaussian view 5.08. The calculated molecular structures in atomic coordinates are shown in Figure 4. Results show that the B3LYP/6-31G+(d,p) DFT model has obvious advantages in geometric optimization of the molecular structure, and there was no imaginary frequency in all calculations. Therefore, stable molecular conformations can be obtained. These obtained atomic coordinates were then used as the input to the calculation of the vibration modes that caused the resonant frequencies.

#### 2.4.2. Comparison of Experimental and Theoretical Spectra

**Figure 5:**
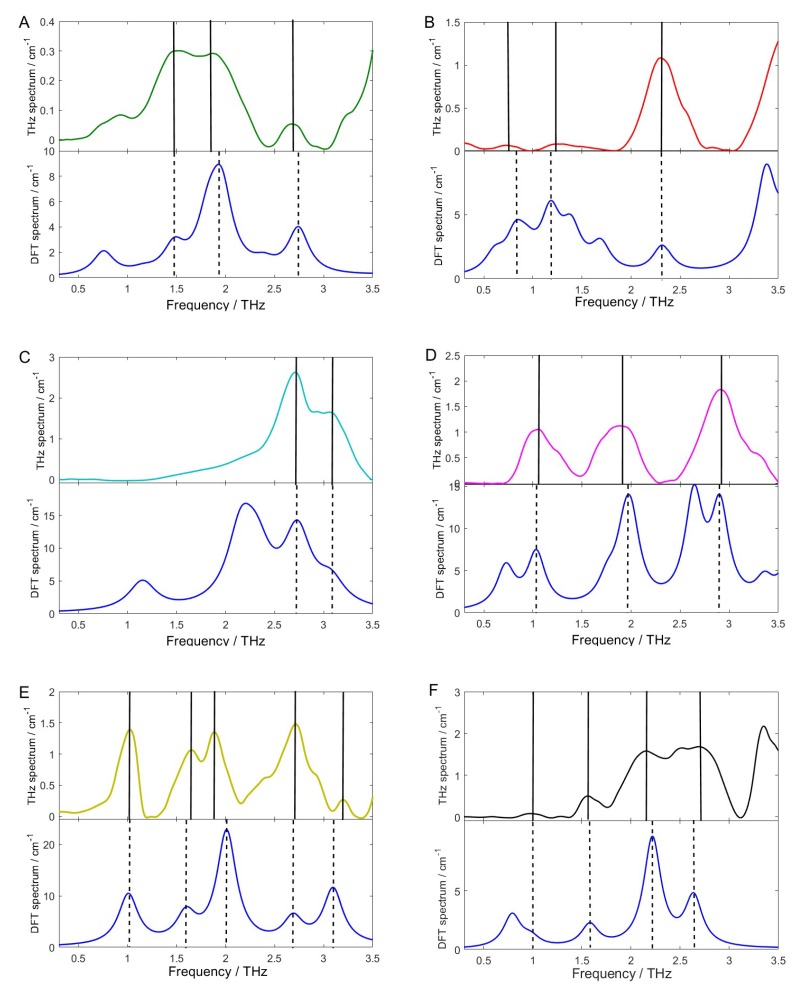
Comparison of the experimental and theoretical spectra of the pesticides (**A**) chlorpyrifos; (**B**) fipronil; (**C**) carbofuran; (**D**) dimethoate; (**E**) methomyl, and (**F**) thidiazuron.

Based on the calculated atomic coordinates and the optimized molecular structures, the theoretical absorption spectra of the isolated molecule of the six pesticides were simulated. Figure 5 shows the comparison between the theoretical spectra simulated by B3LYP/6-31G+(d,p) DFT model and the experimental spectra processed by WTD and SBC. It can be seen that the experimental measured spectra processed by WTD + SBC were in reliable agreement with the corresponding DFT theoretical simulated spectra except with slight frequency shift and few absorption peaks missing. The relatively high spectral similarity between the theoretical and the experimental spectrum indicates that the preprocessing method of WTD + SBC can improve the analytical resolution accuracy of the THz absorption peaks. The discrepancy between the experimental and theoretical spectra is mainly due to the different state of the tested sample, because the experimental samples were prepared pellets of solid powders, while the DFT simulations were based on the isolated molecules. Therefore, the intermolecular interaction, crystal field effect, and crystal resonance were not included in theoretical simulation. Furthermore, the experiment was carried out at laboratory temperature (294 K), but the simulation was based on a temperature of 0 K, so the thermal effect was ignored. The number of the theoretical absorption peaks is larger than that of the experimental absorption peaks. This may be due to the limitation of THz experimental instruments, resulting in some molecular vibration modes that cause absorption peaks unable to be detected.

#### 2.4.3. Assignment of Absorption Peaks

The formation mechanism of the THz characteristic absorption peaks can be assigned and analyzed using the visualization function in GaussView 5.08 (Gaussian Inc., Wallingford, CT, USA). Table 2 lists the assignment of the vibration modes that caused peaks according to the DFT simulated results. It can be explained that the absorption peak of chlorpyrifos at 1.47 THz is generated by the out-plane bending vibration of C-C (15 C and 25 C) and C-C (18 C and 21 C). The peak at 1.99 THz was caused by the interaction of the in-plane stretching vibration of P=O (11 P and 13 O) and the out-plane bending vibration of C-C (15 C and 25 C) and C-C (18 C and 21 C). The peak at 2.71 THz was assigned as in-plane stretching vibration of P=O (11 P and 13 O). The absorption peaks of fipronil at 0.76, 2.31, and 3.61 THz were all generated by the in-plane bending vibration of C-N (3 C and 15 N). The absorption peaks of carbofuran at 2.72 and 3.06 THz were all formed by the in-plane bending vibration of C-O (24 C and 10 O). For the molecule of dimethoate, its absorption peak at 1.05 THz was caused by the in-plane bending vibration of C-C (9 C and 7 C). Its peak at 1.89 THz was generated by the interaction of the in-plane bending vibration of C-N (3 C and 1 N) and the out-plane bending vibration of O-C (15 O and 17 C) and O-C (16 O and 21 C). The peak at 2.92 THz was generated by the interaction of the in-plane bending vibration of C-S (9 C and 12 S) and the out-plane bending vibration of C=O (7 C and 8 O). For the molecule of methomyl, the absorption peaks at 1.01 and 1.65 THz were all generated by the in-plane bending vibration of N-C (1 N and 5 C). The peak at 1.91 THz was formed by the interaction of the in-plane bending vibration of C-N (3 C and 1 N) and the out-plane bending vibration of S-C (16 S and 17 C). The peaks at 2.72 and 3.20 THz were caused by the in-plane bending vibration of C-H (5 C and 6 H) and C-O (3 C and 9 O), respectively. For the molecule of thidiazuron, the absorption peak at the 0.99 THz was formed by the π-bond stretching vibration of benzene ring structure. The peaks at 1.57, 2.17, and 2.66 THz were formed by the in-plane bending vibration of N-C (15 N and 14 C), C=O (14 C and 15 O), and N-C-N (15 N, 14 C and 12 N), respectively. The formation mechanisms of THz experimental absorption peaks were assigned and identified based on the MDS of DFT, which lay a foundation for the application of THz technology in the detection of pesticides.

## 3. Materials and Methods 

### 3.1. Sample Preparation

The six kinds of pesticide standard substances measured in this experiment were purchased from Sigma-Aldrich (Sigma-Aldrich Co., St. Louis, MO, USA) and used without further purification. Table 3 lists the physicochemical properties of the chlorpyrifos, fipronil, carbofuran, dimethoate, methomyl, and thidiazuron. These pesticide standard substances were in state of solid powder (analytic grade ≥ 99.0%) and homogenized in agate mortar, sieved with 100 mesh, mixed with PE powder (Sigma-Aldrich) in proportion to 1:1, and suppressed for 4 min under pressure of 30 MPa. The pesticide samples were prepared in state of disc-shaped pellets with diameters of 13 mm and thicknesses of 1.66, 1.69, 1.32, 1.79, 1.59, and 1.57 mm, respectively. The two surfaces of the pellets should be smooth and parallel to reduce the effect of scattering loss [30].

### 3.2. THz Spectral Acquisition

The THz absorption coefficient and refractive index spectra in the frequencies of 0.1–3.5 THz were obtained with the TeraPulse 4000 THz-TDS system, Inc (Teraview, UK). TeraPulse 4000 consists of ultrashort pulse fiber laser, laser gated photo-conductive semiconductor emitter, and laser gated photo-conductive semiconductor receiver. It uses the ultra-fast fiber laser sources and semiconductor-based detection systems [31,32]. The central wavelength of the ultrashort pulse fiber laser is 780 nm, the pulse width is 90 fs, and the scanning precision is 50–150 um. Experiments were conducted at room temperature of 294 K, and dry nitrogen was filled into the sample bin to avoid the influence of moisture. When acquiring the THz spectra, THz pulse focused on the sample pellet vertically. The average spectrum of 900 time-domain scans with PE as reference is obtained as the spectrum of the tested sample. PE is an ideal mixture because it has extremely low absorption of THz radiation and therefore has no effect on the location of absorption peaks of the pesticides. 

### 3.3. THz Spectral Processing

The THz spectral signals can be influenced by the ambient temperature, resistance, thermal noise, and sampling state. These factors will cause spectral noise and baseline drift, thereby reducing the absorption intensity and SNR of the spectrum [33]. Therefore, it is necessary to preprocess spectral data to eliminate various uncertainties and enhance the useful information of spectrum. To reduce the spectral noise, WTD algorithm is used. It is supposed that the spectrum consists of the useful signal and the noise. Firstly, by decomposing the spectrum into several levels, the calculated wavelet coefficients of the signals are large, while those of the noises are small. Therefore, a threshold function can be used to separate the wavelet coefficients of the signals and the noises. Finally, the de-noised spectrum can be obtained by reconstructing the wavelet coefficients of the signals [34]. PSNR and RMSE are used to evaluate the de-noising effect. PSNR and RMSE are expressed as follows [35]:

(1) $$RMSE=\sqrt{\frac{{\sum_{t=1}^{N}\left[f(t)-\overset{\wedge }{f}(t)\right]}^{2}}{N}}$$

(2) $$PSNR=10\;\log_{10}(\frac{\max|f(t)|}{RMSE})$$

where *f*(*t*) and $\overset{\wedge }{f}(t)$ are the original signal and the de-noised signal respectively. *N* is the signal length. max|f(t)| is the intensity value of the maximum peak in the original signal. The smaller the RMSE value and the larger the PSNR value are, the better the signal de-noising performance is.

It is a common problem that there are baselines drifts for the collected THz spectra, which is caused by the high frequency absorption. The baseline is composed of absorption features superimposed upon a continuous and slowly varying background. It varies greatly among different spectra, even for the similar samples. The inconsistent baseline drifts will hamper the interpretation of the spectra, especially in the quantitative analysis. Therefore, it is necessary to remove the baseline drifts in THz spectra. SBC algorithm is an efficient tool to remove the baseline drift in spectroscopic analysis. It estimates the baseline based on the asymmetric least squares smoothing as follows [36]:

(3) $$z=\arg\min\left\{\sum_{i=1}^{n}w_{i}{\left(y_{i}-z_{i}\right)}^{2}+\mu \sum_{i=1}^{n}\left(\Delta^{2}z_{i}{}^{2}\right)\right\}$$

where *z* is the estimated baseline; *y* is the original spectrum; *w* is the weight chosen asymmetrically. If *y* > *z*, *w* = *p*, otherwise, *w* = 1 − *p*, and *p* is the weight parameter; ∆ is a difference operator used for spectral smoothing; *μ* is a regularization parameter used for fitting error. *i* = 1, 2, …, *n*, *n* is the length of the spectrum. The estimated baseline is removed from the original spectrum to pull the baseline drifts back to zero absorbance.

### 3.4. Density Functional Theory

Density functional theory (DFT) has been widely used in physics and chemistry as a theoretical tool for calculating molecular energy and properties. Electronic structures and energy calculations based on DFT and computer simulations based on molecular dynamics have greatly contributed to the understanding of the microscopic materials [37]. DFT calculations are able to provide accurate and approximate description of the chemical bonds in molecules. They can be used to predict the equilibrium and nonequilibrium properties of condensed systems. Furthermore, they can be used to study the large scaled and disordered systems and the interatomic forces for molecular dynamics simulations (MDS) [38]. Based on the idea that the electron density is the fundamental quantity for describing atomic and molecular ground states, Parr and Yang have given sharp definitions for chemical concepts in various branches of chemistry. Becke three-parameter Lee-Yang-Parr (B3LYP) functional in combination with various basis sets has been extensively used for calculating molecular geometries, vibrational frequencies, ionization energies and electron affinities, dipole and quadrupole moments, atomic charges, infrared intensities, and magnetic properties [39].

## 4. Conclusions

This work presents an analytical strategy for studying fingerprint absorption characteristics of six pesticides using THz-TDS. THz spectroscopy evidences the spectral characteristics of chlorpyrifos, fipronil, carbofuran, dimethoate, methomyl, and thidiazuron, reiterating its potential in characterization and identification of pesticides. For spectral processing and improving spectral SNR, WTD method based on db5 wavelet function, 5-layer decomposition, and soft-threshold was used to eliminate spectral noise. In addition, SBC method based on asymmetric least squares smoothing was used to remove spectral baseline drift. Results show that WTD and SBC are effective processing methods for THz spectra. The density functional models of B3LYP/6-31G+(d,p) in DFT were demonstrated to be competent for pesticides molecular geometric configurations and dynamics simulations. Results show that there is a good match between the THz spectra and DFT spectra. Therefore, the formation mechanism of pesticide absorption peaks in THz spectra can be identified and assigned according to the theoretical spectra of DFT. In this work, the molecular fingerprint characteristics of several kinds of pesticides were studied by using THz-TDS technology, which provides the theoretical and experimental basis for the detection of pesticide residues in agricultural products. 

## Figures and Tables

**Table 1:** Wavelet threshold de-noising (WTD) thresholds and evaluations of spectral de-noising.

Pesticides	Thresholds					Evaluations	
	Level-1	Level-2	Level-3	Level-4	Level-5	PSNR	RMSE
Chlorpyrifos	0.037	0.120	0.150	0.197	0.052	39.330	0.026
Fipronil	0.018	0.099	0.118	0.260	0.227	36.865	0.036
Carbofuran	0.048	0.432	0.692	0.472	1.548	35.821	0.107
Dimethoate	0.714	1.064	0.893	1.225	1.441	32.502	0.173
Methomyl	0.428	0.877	0.986	1.622	0.074	33.088	0.159
Thidiazuron	1.172	0.784	0.781	0.568	0.054	32.553	0.124

**Table 2:** Assignment of the absorption peaks.

DFT Simulation (THz)	THz Experiment (THz)	Shift (THz)	Vibration Modes
Chlorpyrifos			
1.47	1.47	0	δ(C-C)oop
1.90	1.93	−0.03	υ(P=O)ip + δ(C-C)oop
2.74	2.73	0.01	υ(P=O)ip
Fipronil			
0.82	0.76	0.06	δ(C-N)ip
1.18	1.23	−0.05	δ(C-N)ip
1.36	-	-	δ(C-N)ip + δ(C-S)oop
1.67	-	-	δ(C-N)ip + δ(C-S)oop
2.31	2.31	0	δ(C-N)ip
Carbofuran			
1.15	-	-	δ(C-N)ip
2.20	-	-	δ(C-N)ip + υ_breathe_
2.72	2.72	0	δ(C-O)ip
3.06	3.06	0	δ(C-O)ip
Dimethoate			
0.72	-	-	δ(C-C)ip
1.03	1.05	−0.02	δ(C-C)ip
1.93	1.89	0.04	δ(C-N)ip + δ(C-O)oop
2.64	-	-	δ(C-S)ip + δ(C-O)oop
2.90	2.92	−0.02	δ(C-S)ip + δ(C-O)oop
Methomyl			
1.01	1.01	0	δ(C-N)ip
1.59	1.65	−0.06	δ(C-N)ip
2.01	1.91	0.10	δ(C-N)ip + δ(C-S)oop
2.68	2.72	−0.04	δ(C-H)ip
3.09	3.20	−0.11	δ(C-O)ip
Thidiazuron			
0.98	0.99	−0.01	υ_breathe_
1.58	1.57	0.01	δ(C-N)ip
2.21	2.17	0.04	δ(C-O)oop
2.63	2.66	-0.03	δ(N-C-N)oop

υ: stretching vibration, δ: bending vibration, oop: out-plane bending, ip: in-plane bending.

**Table 3:** Physicochemical properties of the pesticides.

Pesticides	CAS Number	Molecular Formula	Molecular Mass	Molecular Structure
Chlorpyrifos	2921-88-2	C_9_H_11_Cl_3_NO_3_PS	350.59	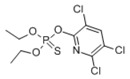
Fipronil	120068-37-3	C_12_H_4_Cl_2_F_6_N_4_OS	437.2	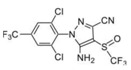
Carbofuran	1563-66-2	C_12_H_15_NO_3_	221.25	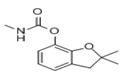
Dimethoate	60-51-5	C_5_H_12_NO_3_PS_2_	229.12	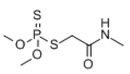
Methomyl	16752-77-5	C_5_H_10_N_2_O_2_S	162.23	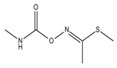
Thidiazuron	51707-55-2	C_9_H_8_N_4_OS	220.2	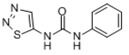

## References

[1] Siegel P.H. (2004). Terahertz technology in biology and medicine. IEEE Trans. Microw. Theory Tech..

[2] Naftaly M., Miles R.E. (2007). Terahertz time-domain spectroscopy for material characterization. Proc. IEEE.

[3] Mccrindle I.J.H., Grant J., Drysdale T.D., Cumming D.R.S. (2014). Multi-spectral materials: Hybridisation of optical plasmonic filters and a terahertz metamaterial absorber. Adv. Opt. Mater..

[4] Popov A.K., Myslivets S.A. (2017). Generation, amplification, frequency conversion, and reversal of propagation of THz photons in nonlinear hyperbolic metamaterial. Opt. Lett..

[5] Mizuno M., Fukunaga K., Hosako I. (2008). Terahertz spectroscopy and its applications to dielectric and electrical insulating materials. Proceedings of the 2008 International Symposium on Electrical Insulating Materials.

[6] Nie P., Qu F., Lin L., Dong T., He Y., Shao Y., Zhang Y. (2017). Detection of water content in rapeseed leaves using terahertz spectroscopy. Sensors.

[7] Duvillaret L., Garet F., Coutaz J.L. (2002). A reliable method for extraction of material parameters in terahertz time-domain spectroscopy. IEEE J. Sel. Top. Quantum Electron..

[8] De Cumis U.S., Xu J.H., Masini L., Degl'innocenti R., Pingue P., Beltram F., Tredicucci A., Vitiello M.S., Benedetti P.A., Beere H.E. (2012). Terahertz confocal microscopy with a quantum cascade laser source. Opt. Express.

[9] Kawase K., Ogawa Y., Watanabe Y., Inoue H. (2003). Non-destructive terahertz imaging of illicit drugs using spectral fingerprints. Opt. Express.

[10] Neumaier P.F., Schmalz K., Borngräber J., Wylde R., Hübers H.W. (2015). Terahertz gas-phase spectroscopy: chemometrics for security and medical applications. Analyst.

[11] Missori M., Bagniuk J., Clerici M., Lojewska J., Misiti M.C., Peters L., Morandotti R., Conte A.M., Pulci O., Teodonio L. (2015). Terahertz Waves for Ancient Manuscripts Conservation. Proceedings of the 2015 European Conference on Lasers and Electro-Optics-European Quantum Electronics Conference.

[12] Recur B., Lewis D., Darracq F., Balacey H., Sleiman J.B., Guillet J.P., Mounaix P. (2015). Low-frequency noise effect on terahertz tomography using thermal detectors. Appl. Opt..

[13] Pan C., Zhang R., Luo H., Shen H. (2016). Baseline correction of vibration acceleration signals with inconsistent initial velocity and displacement. Adv. Mech. Eng..

[14] Kokkoniemi J., Lehtomäki J., Juntti M. (2016). A discussion on molecular absorption noise in the terahertz band. Nano Commun. Netw..

[15] Blanchard P., Brüning E. (2003). Density Functional Theory of Atoms and Molecules. Mathematical Methods in Physics.

[16] Wiberg K.B. (2015). Comparison of density functional theory models’ ability to reproduce experimental ^13^C-NMR shielding values. J. Comput. Chem..

[17] Witko E.M., Buchanan W.D., Korter T.M. (2011). Terahertz spectroscopy and solid-state density functional theory simulations of the improvised explosive oxidizers potassium nitrate and ammonium nitrate. J. Phys. Chem. A.

[18] Carvalho F.P. (2017). Pesticides, environment, and food safety. Food Energy Security.

[19] Maeng I., Baek S.H., Kim H.Y., Ok G.S., Choi S.W., Chun H.S. (2014). Feasibility of using terahertz spectroscopy to detect seven different pesticides in wheat flour. J. Food Prot..

[20] Baek S.H., Ju H.K., Hwang Y.H., Kang M.O., Kwak K., Chun H.S. (2016). Detection of methomyl, a carbamate insecticide, in food matrices using terahertz time-domain spectroscopy. J. Infrared Millim. Terahertz Waves.

[21] Qin J., Ying Y., Xie L. (2013). The Detection of Agricultural Products and Food Using Terahertz Spectroscopy: A Review. Appl. Spectrosc. Rev..

[22] Rajput A.H., Uitti R.J., Stern W., Laverty W., O’Donnell K., O'Donnell D., Yuen W.K., Dua A. (2016). Geography, drinking water chemistry, pesticides and herbicides and the etiology of Parkinson's disease. Can. J. Neurol. Sci..

[23] Barres B., Micoud A., Corio-Costet M.-F., Debieu D., Fillinger-David S., Walker A.-S., Délye C., Grosman J., Siegwart M. (2016). Trends and challenges in pesticide resistance detection. Trends Plant Sci..

[24] Cen H., Weng H., Yao J., He M., Lv J., Hua S., Li H., He Y. (2017). Chlorophyll fluorescence imaging uncovers photosynthetic fingerprint of citrus huanglongbing. Front. Plant Sci..

[25] Kong W., Zhang C., Huang W., Liu F., He Y. (2018). Application of hyperspectral imaging to detect sclerotinia sclerotiorum on oilseed rape stems. Sensors.

[26] Wu D., Wang S., Wang N., Nie P., He Y., Sun D.W., Yao J. (2013). Application of time series hyperspectral imaging (TS-HSI) for determining water distribution within beef and spectral kinetic analysis during dehydration. Food Bioprocess Technol..

[27] Zhang C., Jiang H., Liu F., He Y. (2016). Application of near-infrared hyperspectral imaging with variable selection methods to determine and visualize caffeine content of coffee beans. Food Bioprocess Technol..

[28] Hua Y., Zhang H. (2010). Qualitative and quantitative detection of pesticides with terahertz time-domain spectroscopy. IEEE Trans. Microw. Theory Tech..

[29] Lee D.K., Kim G., Kim C., Jhon Y.M., Kim J.H., Lee T., Son J.H., Seo M. (2016). Ultrasensitive detection of residual pesticides using THz near-field enhancement. IEEE Trans. Terahertz Sci. Technol..

[30] Authority E.F.S. (2016). Conclusion on the peer review of the pesticide risk assessment of confirmatory data submitted for the active substance haloxyfop-P. Efsa J..

[31] Bauerschmidt S., Preu S., Malzer S., Döhler G.H., Wang L.J., Lu H., Gossard A.C. (2010). Continuous wave Terahertz emitter arrays for spectroscopy and imaging applications. Proceedings of the Terahertz Physics, Devices, and Systems IV: Advanced Applications in Industry and Defense.

[32] Kitamura S. (2015). Terahertz Spectrometry Device and Method, and Nonlinear Optical Crystal Inspection Device and Method. U.S. Patent.

[33] Fan X., Xie W., Jiang W., Yi L., Huang X. (2016). An improved threshold function method for power quality disturbance signal de-noising based on stationary wavelet transform. Trans. China Electrotech. Soc..

[34] Ling Z.B., Wang P.Y., Wan Y.X., Wang Y.Z., Cheng D.F., Tong-Lin L.I. (2016). A combined wavelet transform algorithm used for de-noising magnetotellurics data in the strong human noise. Chin. J. Geophys..

[35] El B’charri O., Latif R., Elmansouri K., Abenaou A., Jenkal W. (2017). ECG signal performance de-noising assessment based on threshold tuning of dual-tree wavelet transform. Biomed. Eng.Online.

[36] Chen X., Parrott E.P.J., Ung S.Y., Pickwell-Macpherson E. (2017). A Robust baseline and reference modification and acquisition algorithm for accurate THz imaging. IEEE Trans. Terahertz Sci. Technol..

[37] Ipatov A., Cordova F., Doriol L.J., Casida M.E. (2017). Excited-state spin-contamination in time-dependent density-functional theory for molecules with open-shell ground states. J. Mol. Struct..

[38] Huang K., Li Y., Tao S., Wei G., Huang Y., Chen D., Wu C. (2016). Purification, characterization and biological activity of polysaccharides from dendrobium officinale. Molecules.

[39] Sepahpour S., Selamat J., Abdul Manap M.Y., Khatib A., Abdull Razis A.F. (2018). Comparative analysis of chemical composition, antioxidant activity and quantitative characterization of some phenolic compounds in selected herbs and spices in different solvent extraction systems. Molecules.

